# Adolescents Engaged in Radicalisation and Terrorism: A Dimensional and Categorical Assessment

**DOI:** 10.3389/fpsyt.2021.774063

**Published:** 2022-01-14

**Authors:** Guillaume Bronsard, David Cohen, Issaga Diallo, Hugues Pellerin, Aurélien Varnoux, Marc-Antoine Podlipski, Priscille Gerardin, Laurent Boyer, Nicolas Campelo

**Affiliations:** ^1^Service de Psychiatrie de l'Enfant et de l'Adolescent, CHRU Brest, Brest, France; ^2^Département de Sciences Humaines et Sociales, EA 7479, UFR médecine et santé UBO, EA 3279 (CEReSS, AMU), Brest, France; ^3^Service de Psychiatrie de l'Enfant et de l'Adolescent, Hôpital Pitié-Salpêtrière, Paris, France; ^4^Institut des Systèmes Intelligents et Robotiques, Sorbonne Université, Paris, France; ^5^Hôpitaux Universitaires Pitié Salpêtrière, Paris, France; ^6^Centre Hospitalier Robert-Ballanger, Aulnay-sous-Bois, France; ^7^Centre Hospitalier du Rouvray, Sotteville-lès-Rouen, France; ^8^Médecine de Santé Publique, CHRU Marseille, Marseille, France; ^9^Laboratoire PCPP-EA 4056, Institut de Psychologie, Université de Paris, Paris, France

**Keywords:** radicalisation, adolescence, psychiatric disorder, empathy, suicidality, delinquency

## Abstract

Since 2010 and the founding of the Islamic State, the radicalisation phenomenon in Europe has involved more adolescents and converts to Islam than in previous Islamist terrorist group movements (e.g., Al-Qaeda). In most cases, these adolescents are “homegrown terrorists,” a challenging difference, as they are in confrontation with their home and societal environment. As a new and emerging phenomenon, radicalisation leads to many questions. Are empathic capacities altered? Are they presenting psychiatric pathologies or suicidal tendencies that explain why they put themselves in serious dangers? Are they just young delinquents who simply met a radical ideology? In January 2018, by special Justice Department authorisation, we contacted all minors (*N* = 31) convicted in France for “criminal association to commit terrorism.” We assessed several sociodemographic, clinical and psychological variables, including empathy and suicidality, in half of them (*N* = 15) and compared them with 101 teenagers convicted for non-terrorist delinquency who were placed in Closed Educational Centres (CEC). The results show that adolescents engaged in radicalisation and terrorism do not have a significant prevalence of psychiatric disorders, suicidal tendencies or lack of empathy. It also appears that they have different psychological profiles than delinquent adolescents. “Radicalised” adolescents show better intellectual skills, insight capacities and coping strategies. In addition, the manifestation of their difficulties is less externalised than adolescents from the CEC, having committed very few delinquent acts.

## Introduction

Radicalisation is a complex phenomenon. Several authors insist on its transformative characteristics and the dangers of overgeneralization (1). Initial research, carried out on different terrorist organisations, in particular Al-Qaida (2), has shown that violent radicalisation involves primarily young men, between 25 and 30 years old on average, from the upper and middle classes; 63% of them had attended universities, and 73% were married and/or had children. Most of them had no criminal history before their radical involvement and exhibited solid mental health (2).

If we focus on the radicalisation phenomenon as it has appeared in Europe, at the same time as the advent of the Islamic State (or Daesh), we can identify several characteristics. France has been particularly concerned with the radicalisation phenomenon compared to other European countries, either in terms of the number of nationals involved or as a target of terrorist attacks (3–7). A total of 5,000 Europeans went to the Iraqi-Syrian conflict zone, including 700 French individuals, and at least 2,500 Europeans fought for Daesh (5). According to some studies, the radicalisation phenomenon would appear particularly prevalent among subjects involved with delinquency during their life and who have presented difficulties at familial, social, and professional integration (4).

However, several observations call these assertions into question. (1) Women have taken on an important role; since 2015; the proportion of women arrested for terrorism-related activities has increased, and one in three French jihadists in the Iraqi-Syrian war zone is a woman (3, 8, 9). (2) The individuals involved in this phenomenon are increasingly young (10, 11). 25% of the subjects reported for radicalisation-related acts in France are minors (12). (3) An increasing number of subjects are using violence to turn against their environment on behalf of an ideology that comes from a geographically distant space (some political specialists call it homegrown-terrorism), which explains the high proportion of converted radicalised individuals (41% in France) among subjects concerned by the radicalisation phenomenon (12, 13). Thus, in Europe during 2015, the characteristics of radicalised individuals were significantly different than those of Al-Qaida members in the 2000s (9). The training groups and the foreign-terrorism dimension gave way to homegrown terrorism that does not necessarily require initiation or an introduction into the radical group, but rather a more personal appropriation of a diffuse ideology available on the internet. We found that for these subjects, a wide range of motivations justified their radical commitment (11).

However, the mainstream press and some public reports (4) suggest that these subjects, who grew up in Europe, are young delinquents who come from disadvantaged and marginalised areas and who only secondarily encounter an ideology that claims violence. Although this assumption contradicts earlier research on terrorist carried out by Al-Qaida (2), it must be explored because of the evolution of radicalisation since 2010 (13). For this purpose, we aimed to compare all French adolescents prosecuted for “criminal association to commit terrorism” (Association de Malfaiteur en vue d'une entreprise **Terroriste**” in French or “AMT”) with a national sample of adolescents convicted of “common” crimes. Subjects involved with radicalisation can easily be perceived as “mad,” “bad” or “sad,” sometimes even in a cumulative and variable way. We specifically determined the prevalence of psychiatric disorders, the ability to empathise and potential depression or suicidal tendency indicators.

## Methodology

### Design

This is a case-control study investigating radicalised adolescents involved in terrorist activities in France and comparing their occurrence of psychiatric diagnosis, their empathic abilities and their suicidal tendencies with adolescents in France convicted of common crimes. The list of all adolescents convicted of AMT in France was obtained by special authorisation from the French Department of Justice in 2018. We obtained ethical authorisation to assess the AMT group from the Ethical Committee (*Comité de Protection des Personnes*) of Strasbourg Hospital (CPP-Est IV in January 2019) and to assess the common delinquency group from the Ethical Committee (*Comité de Protection des Personnes*) of the *Université Aix-Marseille* (n° 2016-09-11-1 on November 10, 2016). Each participant and their parents received a briefing note, and they signed a written consent form to participate in the study. The management of individual and protected data has been reported to the “*Commission Nationale Informatique et Liberté*” (French for “National Commission on Informatics and Liberty”).

### Population

For the group of radicalised adolescents, we kept three principal inclusion criteria: (1) prosecution for “criminal association to commit terrorism” (AMT); (2) a minority at the moment we started our study in January 2018; and (3) being able to speak, read, and write French. Concerning “radicalisation,” we wanted to choose the most objective extreme commitment criteria. Being prosecuted for “criminal association to commit terrorism” is a charge supported by evidence that confirms links with one or more individuals whose ideology preaches the use of violence. When the recruitment of adolescents prosecuted for AMT began, in January 2018, the Youth Judicial Protection Service (“Protection Judiciaire de la Jeunesse” in French or “PJJ”) informed us that 34 subjects met our inclusion criteria in French territory. Three of them were released during the investigation and were thus excluded. Sixteen refused to participate in the study. Therefore, we were able to include and collect complete data from 15 subjects. Even if it does not seem like many, it represents half of a marginal population of French adolescents involved in AMT prosecution in January 2018. We attempted to achieve representativeness in the sample studied by comparing specific objective characteristics from subjects included and from those who refused to participate in the study (e.g., age, gender, sibling composition, proportion of school years repeated, intervention of social services or care pathway). We did not find significant differences between the subjects included and those who refused to participate in the study, which allows us to assert that the sample tested for the study is representative of this population. We met the adolescents where they were detained, in jail or at the closed educational centre (CEC) (“Centre Educatif Fermé” in French) or educational units in an open setting. Consultations were always made by psychologists, child psychiatrists or clinical research associates.

Regarding the group of adolescents with a profile of “common” delinquent, the PJJ identified 18 CECs through the country to ensure sample representativeness (the list is available in the Supplementary Material). The study was proposed to all adolescents between 13 and 17 years old who were placed in these CECs by a judge as an alternative to detention for criminal acts. Five of the 18 CECs contacted did not have enough residents to participate in the study, and four were not able to do so. Therefore, recruitment was carried out in the remaining nine CECs. A total of 113 adolescents agreed to participate in the study, but complete data were not available for 12 of them. In total, we selected a group of 101 adolescents placed in CEC.

### Tools

To analyse the group of radicalised adolescents, we started creating a specific grid for the study. Given the low amount of data in the scientific literature about radicalised individual characteristics in France, this grid was part of an exploratory approach. Various characteristics (e.g., sociocultural, life history, psychopathology, behaviour, speech, commitment reasons, etc.), identified in some studies on this topic (10, 14, 15) were first collected to create this grid (available in Supplementary Material). It was completed by semistructured interviews with participants and, thereafter, in collaboration with assigned educators or with additional information collected from PJJ's educational files. The grid, specific to the radicalisation phenomenon, was not completed with adolescents placed in CECs for common delinquency. So we do not present the comparative results between the two populations.

Afterwards, we evaluated several sociodemographic characteristics for both groups using a specific questionnaire that shows, for example, the family situation of the subject, his life history, and his criminal record. We also evaluated the presence of negative childhood events through the “Adverse Childhood Experience” (ACE) questionnaire. Previous research using this scale showed links between childhood maltreatment and public health problems in adults (16).

Categorical psychiatric diagnoses were mainly evaluated using MINI-KID 2 according to DSM-V and CIM-10 criteria. This semistructured interview is aimed at children and adolescents, but it does not evaluate the presence of psychotic disorders, which is why we added the subscale of the MINI for adults, which is more related to these disorders. Finally, we evaluated the presence of a borderline personality disorder (BPD) using the Ab-DIB (Abbreviated-Diagnostic Interview for Borderline) (17). For intellectual functioning, we evaluated fluid intelligence with Raven Matrices from the WAIS-IV evaluation scale.

Several psychological dimensions were explored. The severity of depression was evaluated using the Adolescent Depression Rating Scale (ADRS). The suicidal risk was evaluated using the Columbia evaluation scale for suicidal risk gravity (C-SSRS), created by Posner et al. using suicidal behaviour definitions from “The Columbia History Form.” Distress was evaluated using the Beck Hopelessness Scale (BHS). Conceived by Beck, Weissman, Lester and Trexler (18), this scale evaluates the intensity of people's pessimism and their negative perception of the future. This questionnaire has been examined among the clinical population (19–24). This scale has predictive value for suicide attempts. Indeed, a high score indicates a better correlation of suicidal intention than the severity of clinical depression. For practical reasons, this scale was not used with adolescents placed on CEC, and for this reason, we could not perform a comparison with the AMT sample.

In addition to exploring depressive and suicidal dimensions, we also evaluated several psychological dimensions that have been associated with psychopathology and/or deviant behaviours. We used the Reasons for Living Inventory for Adolescents (RFL-A). This tool was developed and approved by Osman et al. (25). It evaluates beliefs that lead adolescents to want to live and not to resort to suicidal actions. We also evaluated their sense of empathy using Bryant's child and adolescent empathy scale (26), translated into French in 2008 (27), and their emotional attachment through the inventory of parent and peer attachment (IPPA) from Armsden and Greenberg (28) [French translation by Touch and Sigel, 2006, (29)]. We also evaluated the level of impulsiveness and hostility of these adolescents using a section from the Eysenck I7 questionnaire for children and adolescents (30) and the adolescent version of the Buss-Durkee Hostility Inventory (31). Self-esteem was evaluated with the Rosenberg Self-Esteem Scale (RSES), translated into French by Vallieres and Vallerand (32). Last, coping was evaluated using the Adolescent Coping Scale (ACS). Created and approved in Australia by Frydenberg and Lewis, this scale evaluates specific behaviours used to deal with a situation or to solve a problem. ACS results allow us to evaluate the recurrence of using coping strategies, divided into productive coping strategies, coping strategies that require external support, unproductive coping strategies, and less effective strategies to deal with and solve difficulties (33).

### Statistics

Statistical analyses were performed using R software 4.0.2 by resorting to two-tailed tests with a level of significance fixed at 5%. First, we described the variables in AMT and CEF samples. Quantitative variable distributions were summarised using the mean, standard deviation, and min-max. Alternatively, we used the median, q1-q3 and min-max, depending on the shape of the distribution. Qualitative variable distributions were summarised using the number and percentage of occurrences. Second, we compared the variables between the AMT and CEC samples. Depending on the validity of the assumptions, quantitative variables were either compared using Welch *t*-tests or Wilcoxon rank-sum tests. Similarly, qualitative variables were either compared using chi-squared tests without continuity correction or Fisher's exact tests.

## Results

### Characteristics of AMT Adolescents (*N* = 15)

The AMT group was composed of six girls and nine boys, with an average age of 17 years. In terms of family environment, 5 (33.3%) of them had poor socio-economic backgrounds, 12 (80%) of them went through either a bereavement or a sudden separation during their life, seven (46.7%) of them expressed that they suffered a sort of negligence during childhood, nine (60%) of them notified social care interventions, and five (33.3%) of them confirmed that they experienced family socioeconomic insecurity. Last, four (26.7%) of them reported that household members' had been imprisoned. Academically, only five (33.3%) of these adolescents had enough difficulties to repeat a year, which is a common practise in France. Otherwise, they had unremarkable results on the fluid intelligence tests (Matrices); all had average intelligence, 11 (73.3%) adolescents were in the core and four (26.7%) in the low core. Surprisingly, given their having been prosecuted, a large majority of them expressed a desire to work in personal care jobs (childcare assistant, nurse, specialised educator or doctor).

Table 1 summarises the religious sphere, origins and migration status, and views about marginalisation or political ideology for each AMT individual and his/her family. Most adolescents were born in France mostly from migrants or mixed parents. The sample included three (20%) adolescents who converted to Islam, while the others came from families with established Muslim belief traditions. When we met these adolescents, they all assured us they were believers and Muslims. Even if none of them had faithfully practised any religion before the ideological adherence, seven (46.7%) of them affirm that they now do. Additionally, the ACS results show that resorting to spirituality is the most frequent coping strategy used to face difficulties they encounter in their everyday life. Regarding feelings of marginalisation, we distinguished whether the adolescent and/or his/her family expressed feelings of being marginalised or not, and whether they did not share similar views. We found that one third of AMT adolescents (all females) expressed no such feelings or political discourse for themselves or their families, one third had such feelings that contrasted with their own families, and one third shared these feeling with their families.

**Table 1:** Parental origins, Muslim beliefs, and feelings of marginalisation of individuals convicted for “criminal association to commit terrorism.”

**Subject**	**Father**	**Mother**	**Parents**	**Feelings of marginalisation**
**1** Born in France	Maghreb (Muslim)	Maghreb (Muslim)	Migrants	No politization or ideological belief. Radicalization through one influencing person.
**2** Born in France	French (non-Muslim)	French (non-Muslim)	French Caucasian	Politization and marginalisation ideas but opposing family discourse
**3** Born in France	Maghreb (Muslim)	Maghreb (Muslim)	Migrants	No politization or ideological belief. Radicalization through one influencing person.
**4** Born in France	Maghreb (Muslim)	Maghreb (Muslim)	Migrants	Politization and marginalisation ideas shared with one parent or both
**5** Born in Maghreb	Maghreb (Muslim)	Maghreb (Muslim)	Migrants	Politization and marginalisation ideas shared with one parent or both
**6** Born in France	Maghreb (Muslim)	Maghreb (Muslim)	Migrants	Politization and marginalisation ideas shared with one parent or both
**7** Born in France	Maghreb (Muslim)	Maghreb (Muslim)	Migrants	Politization and marginalisation ideas but opposing family discourse
**8** Born in France	French (convert to Islam)	Sub-Saharan (Muslim)	Mixed	Politization and marginalisation ideas shared with one parent or both
**9** Born in France	Maghreb (Muslim)	Maghreb (Muslim)	Migrants	Unknown
**10** Born in France	Maghreb (Muslim)	Maghreb (Muslim)	Migrants	Politization and marginalisation ideas but opposing family discourse
**11** Born in France	Sub-Saharan (non-Muslim)	French Caribbean (convert to Islam)	Mixed	Politization and marginalisation ideas shared with one parent or both
**12** Born in France	Maghreb (Muslim convert to Jehovah Christians)	Maghreb (Muslim convert to Jehovah Christians)	Migrants	No politization or ideological belief. Radicalization through one influencing person.
**13** Born in France	Sub-Saharan (Muslim)	Sub-Saharan (Muslim)	Migrants	Unknown
**14** Born in France	French (non-Muslim)	French (non-Muslim)	French Caucasian	No politization or ideological belief. Radicalization through one influencing person.
**15** Born in France	Maghreb (Muslim)	French (non-Muslim)	Mixed	Politization and marginalisation ideas but opposing family discourse

Concerning their medical history, six (40%) of them were scheduled to participate in psychological follow-up, and three of these were currently under psychiatric care. The psychiatric examinations did not show axis 1 disorders for most of them (*N* = 10, 66.7%). For the other five adolescents, we found comorbid associations with the following diagnoses: borderline personality disorder (BPD), generalised anxiety disorders, conduct disorder (CD) and depression (details are given in Table 2). Suicidal thoughts were observed for only two adolescents, and one of them had attempted suicide. Last, the empathy scale results are at the core of what is expected for adolescents of their age.

**Table 2:** Adolescents engaged in radicalisation and terrorism activities (*N* = 15) compared to adolescents engaged in other delinquent acts (*n* = 101) in 2018 in France.

	**Radicalised adolescents (*N* = 15)**	**Non-radicalised adolescents (*N* = 101)**	**Test**	***P***
**Sociodemographics**				
Age: mean (SD)	16.93 (1.03)	15.93 (1.06)	W	0.001
Female: N (%)	6 (40%)	5 (5%)	F	0.001
Male: N (%)	9 (60%)	96 (95%)		
Siblings in the family: mean (SD) [range]	4.33 (2.06) [0–7]	3.59 (1.6) [0–9]	W	0.128
Living with both parents: N (%)	3 (20%)	28 (28%)	F	0.515
Living with one parent and a stepparent: N (%)	1 (6.7%)	5 (5%)		
Living with one parent: N (%)	7 (46.7%)	41 (40%)		
Other: N (%)	4 (26.7%)	19 (19%)		
Undetermined: N (%)	0 (0%)	8 (8%)		
Repeated grade at school: N (%)^*^	5 (33.3%)	53 (56.4%)	Chi2	0.097
Placement in social services: N (%)	9 (60%)	64 (70.3%)	F	0.548
**Participants' medical history**				
Special education needs for behavioural problems: N (%)	2 (13.3%)	25 (28.4%)	F	0.343
Special education needs for intellectual disability: N (%)	0 (0%)	3 (3.4%)	F	1
Recognition of psychiatric handicap: N (%)	1 (6.7%)	25 (29.1%)	F	0.107
Serious medical comorbidity: N (%)	1 (6.7%)	10 (10.9%)	F	1
Serious psychiatric comorbidity: N (%)	1 (6.7%)	13 (14%)	F	0.687
Psychiatric care in first line institutions: N (%)	3 (20%)	25 (29.4%)	F	0.548
Day care hospital: N (%)	2 (13.3%)	4 (4.6%)	F	0.213
Support in “Maison Des Adolescents” (youth healthcare facilities): N (%)	0 (0%)	10 (11.4%)	F	0.351
Medication for sleep. anxiety. depression or behavioural problems: N (%)	3 (20%)	38 (40.9%)	Chi2	0.122
Acute hospitalisation: N (%)	6 (40%)	16 (17.2%)	F	0.077
Psychiatric hospitalisation: N (%)	3 (20%)	16 (17.2%)	F	0.725
Parents' medical history: N (%)	3 (20%)	27 (31%)	F	0.543
Parents' psychiatric history: N (%)	0 (0%)	10 (11.9%)	F	0.352
**Participants' life history**				
Negative life events during childhood: N (%)				
Emotional violence	6 (40%)	19 (21.8%)	F	0.19
Physical violence	6 (40%)	27 (31%)	F	0.555
Sexual violence	1 (6.7%)	9 (10.3%)	F	1
Emotional neglect	7 (46.7%)	19 (21.8%)	F	0.056
Physical neglect	0 (0%)	8 (9.2%)	F	0.595
Absence of one parent	2 (13.3%)	40 (46%)	Chi2	0.026
Mother being maltreated	2 (13.3%)	16 (19.5%)	F	1
Parental drug abuse	2 (13.3%)	22 (26.8%)	F	0.506
Parental psychiatric condition	1 (6.7%)	24 (29.3%)	F	0.105
One close relative in prison	4 (26.7%)	45 (56.3%)	Chi2	0.056
Personal forensic history: N (%)				
Aggression of a person	2 (13.3%)	70 (76.1%)	F	<0.001
Property damage	0 (0%)	59 (67.8%)	Chi2	<0.001
Drug trafficking	0 (0%)	35 (40.7%)	Chi2	0.002
**Psychiatric assessment**				
No disorder: N (%)	10 (66.7%)	9 (9.8%)	Chi2	<0.001
Major depressive episode: N (%)	3 (20%)	7 (7.6%)	F	0.146
Hypomania (lifetime): N (%)	1 (6.7%)	9 (9.8%)	F	1
Mania (lifetime): N (%)	1 (6.7%)	21 (23.9%)	F	0.187
Agoraphobia: N (%)	1 (6.7%)	8 (8.6%)	F	1
Social phobia: N (%)	0 (0%)	2 (2.2%)	F	1
Obsessive compulsive disorder: N (%)	1 (6.7%)	3 (3.3%)	F	0.438
Panic disorder: N (%)	0 (0%)	1 (1.1%)	F	1
Generalised anxiety disorder: N (%)	4 (26.7%)	11 (12%)	F	0.22
Tourette syndrome: N (%)	0 (0%)	2 (2.2%)	F	1
Conduct disorder: N (%)	3 (20%)	75 (81.5%)	F	<0.001
Anorexia: N (%)	1 (6.7%)	3 (3%)	F	0.473
Post-traumatic stress disorder: N (%)	1 (6.7%)	5 (5.4%)	F	1
Attention deficit/hyperactivity disorder, mix: N (%)	1 (6.7%)	5 (5.6%)	F	1
Attention deficit/hyperactivity disorder, inattention: N (%)	0 (0%)	11 (12%)	F	0.357
Attention deficit/hyperactivity disorder, hyperactivity: N (%)	0 (0%)	9 (9,8%)	F	0.354
Psychotic episode (lifetime): N (%)	2 (13,3%)	12 (13%)	F	1
Mood disorder with psychotic features: N (%)	0 (0%)	4 (4%)	F	1
Borderline personality disorder: N (%)	4 (26.7%)	25 (33.3%)	F	0.766
Ab-DIB score: mean (SD) [range]	9.40 (9.24) [0–34]	11.68 (8.40) [0–43]	W	0.268
**Suicidality**				
ADRS score > 7 (moderate depression): N (%)	1 (6.7%)	9 (12%)	F	1
Suicidal ideation: N (%)				
No	13 (86.7%)	89 (88%)	F	1
Passive	1 (6.7%)	3 (3%)		
Active	1 (6.7%)	9 (8.9%)		
Suicidal attempt: N (%)	1 (6.7%)	6 (7%)	F	1
Reasons for living scores: mean (SD)				
Family alliance	5.31 (0.78)	4.82 (1.31)	W	0.271
Suicide-related concerns	5.05 (1.40)	4.36 (1.65)	W	0.125
Self-acceptance	5.33 (0.71)	4.85 (1.26)	W	0.225
Peer acceptance and support	4.71 (0.97)	4.27 (1.30)	W	0.288
Future optimism	5.11 (0.57)	4.68 (1.37)	W	0.753
Total	5.12 (0.78)	4.61 (1.22)	W	0.191
**Other psychological dimensions**				
Intelligence (Raven): N (%)				
>120	0 (0%)	1 (1.4%)	F	0.032
110 < IQ <120	0 (0%)	3 (4.3%)		
90 < IQ <110	11 (73.3%)	24 (34.8%)		
80 < IQ90	4 (26.7%)	14 (20.3%)		
70 < IQ <800	0 (0%)	12 (17,4%)		
<70	0 (0%)	15 (21.7%)		
Empathy score: mean (SD)	14.38 (4.52)	10.29 (3.32)	W	0.004
Attachment with the mother: mean (SD)				
Confidence	39.47 (8.10)	36.66 (6.96)	W	0.059
Communication	33.29 (6.64)	32.54 (6.78)	W	0.824
Alienation	12.46 (5.87)	22.43 (4.52)	W	<0.001
Attachment with the father: mean (SD)				
Confidence	35.67 (11.78)	34.49 (8.80)	W	0.435
Communication	26.67 (10.42)	29.08 (7.97)	W	0.484
Alienation	14.67 (6.50)	20.14 (4.45)	W	0.002
Attachment with peers: mean (SD)				
Confidence	40.67 (7.63)	37.43 (6.32)	W	0.085
Communication	30.20 (5.89)	26.18 (7.04)	W	0.034
Alienation	15.33 (4.59)	23.29 (3.65)	W	<0.001
Impulsivity: mean (SD)	8.43 (4.29)	13.66 (4.34)	W	<0.001
Hostility: mean (SD)	29.21 (22.45)	42.58 (20.11)	W	0.017
Self-esteem: mean (SD)	32.13 (5.22)	32.78 (4.54)	W	0.641
**Coping scores: mean (SD)**				
Reference to others (total)				
Social action	40.00 (20.29)	32.69 (17.04)	W	0.19
Invest in close friends	58.00 (20.50)	55.81 (22.85)	W	0.691
Seek spiritual support	81.79 (17.93)	45.44 (25.87)	W	<0.001
Seek social support	64.00 (15.92)	42.03 (17.61)	W	<0.001
Seek professional help	54.64 (23.57)	41.74 (21.81)	W	0.059
Productive coping (total)				
Focus on solving problem	70.57 (15.74)	55.42 (20.10)	W	0.013
Work hard and achieve	72.00 (13.94)	56.14 (18.46)	W	0.004
Focus on the positive	76.07 (16.66)	61.67 (20.10)	W	0.017
Seek relaxing diversions	74.00 (18.36)	70.32 (24.01)	W	0.674
Physical recreation	61.00 (25.82)	65.27 (24.97)	W	0.588
Unproductive coping (total)				
Worry	62.29 (16.05)	51.38 (21.75)	W	0.067
Seek to belong	58.00 (10.84)	46.63 (16.46)	W	0.006
Wishful thinking	43.14 (16.39)	40.94 (16.15)	W	0.7
Not coping	42.00 (15.19)	35.52 (13.45)	W	0.14
Tension reduction	36.29 (10.34)	47.56 (17.21)	W	0.023
Ignore the problem	46.43 (17.48)	48.15 (20.96)	W	0.887
Self-blame	57.14 (18.16)	44.85 (18.67)	W	0.037
Keep to oneself	62.86 (22.51)	56.52 (18.56)	W	0.23

*SD, standard deviation; Ab-DIB, abbreviated-diagnostic interview for borderline; ADRS, adolescent depression rating scale*.

* *Repeating a grade at school is a common practise in France*.

Last, their radical commitment was mostly developed on the internet, as only four (26.7%) adolescents had had their first physical contact with individuals professing radical ideology. Most of them mentioned one person they especially believed to be linked with their ideological adherence. Among evident reasons for their ideological adherence, we mostly observed altruistic motivations such as helping the Syrian people (14, 93.3%), reaching an ideal society (13, 86.7%), feelings of injustice or discrimination (11, 73.3%) or running away from their living environment (9, 60%). More rarely, we found attraction to armed struggle (6, 40%), a search for “adrenaline” (5, 33.3%), seeking a clearer distinction between the roles of women and men (5, 33.3%), and fear of hell or a desire to reach heaven (4, 26.7%). All these adolescents joined an ideology that advocates the use of violence in the name of Islam. Finally, 7 (46.7%) of them accomplished plans they first put into words within the ideological group (try to join Daesh: *N* = 4, 26.7%; unfinished knife attack, because they changed their mind at the last minute: *N* = 2, 13.3%; and knife attack against a police officer: *N* = 1, 6.7%).

### Comparison Between AMT Prosecuted Adolescents and Adolescents Placed in CEC

Table 2 summarises the main characteristics of radicalised adolescents (AMT group) vs. “common” delinquent adolescents (CEC group). From a sociodemographic perspective, radicalised adolescents were older, and the proportion of girls was significantly higher than that in the CEC group. The loss of a biological parent, caused by divorce or abandonment, is less common in radicalised adolescents than in CEC adolescents, as well as the imprisonment of household members.

Concerning educational background, repeating a grade is significantly lower among the radicalised adolescents than among those in CECs. Qualitative results show that these repeats are often due to academic disruptions resulting from the radicalisation process. In addition, radicalised adolescents have a much higher cognitive level than CEC adolescents; 20% of the latter present fluid intelligence corresponding to a mild intellectual disability.

Psychiatric assessment results revealed more recurrent disorders in adolescents detained at a CEC, with two significant differences: (1) the lack of mental disorder was much more common in the AMT group (67% vs. 9%) and (2) there were far fewer conduct disorders (CD) in the AMT group (20% vs. 81.5%). Thus, CEC adolescents present much more CD and, in general terms, more psychiatric comorbidities than radicalised adolescents (see details in Table 1). We also noticed a particularly weak suicidal symptomatology proportion in both samples. However, the number of hospitalisations due to a crisis is slightly higher in the AMT group.

The important presence of CD in CEC adolescents is consistent with their preceding indictments, since CEC placement is given to repeat offenders whose acts partially correspond to the definition of CD. These CDs are almost non-existent in radicalised adolescents (except for the AMT indictment). This observation also seems consistent with the hostility level (BDHI) towards others, which is clearly greater in CEC adolescents, similar to the obtained results on the impulsiveness scale (Eysenck). Bryant's empathy scale results also go in this direction, as radicalised adolescents obtain much higher empathy scores than CEC adolescents. They also present more balanced attachment results, as we notice much higher levels of alienation to attachment figures among the CECF adolescents (Table 2).

Coping strategies are also very different in both groups. On the one hand, radicalised adolescents have more resources with which to face their difficulties than do CEC adolescents, as they use much more productive coping strategies. They also more often turn to spirituality, to seeking social action, and to unproductive coping strategies that require a greater capacity for insight (self-blame, worry, seek to belong) in comparison to CEC adolescents. On the other hand, adolescents in CECs willingly turn to unproductive coping strategies, in particular to release their internal stress and reduce tension through physical activity or drug/alcohol abuse.

## Discussion

The prevalence of psychiatric disorders is still in a minority of the adolescents with AMT, even if it is double the usual proportion reported in the general population (34). Furthermore, the distribution of different disorders remains homogeneous and predictable for adolescents of this age, which shows that there is no psychiatric pathology specific to the radicalisation phenomenon, except for a slight overrepresentation of anxiety and depressive disorders and borderline personality disorders. We also noticed a weak proportion of suicidal symptomatology. If we make a comparison with existing results in the literature, we observe that the prevalence of suicide attempts in these adolescents is only slightly higher than in a French adolescent control sample (3.1%), very slightly lower than in dysthymic adolescents (9.5%), and clearly lower than in adolescents presenting a major depressive episode (22.5%) [e.g., (35)]. Compared to a large sample (*N* = 300) of French adolescents hospitalised after a suicide attempt (36), we can see that adolescents with radicalised AMT clearly show fewer depressive disorders or suicidal thoughts. Last, rates of empathy reached by adolescents with AMT place them perfectly at the expected levels for adolescents of their age (26). It is relevant to add that they have a very low rate of conduct disorders and that their previous indictments (apart from AMT) are completely marginal, which rebuts the theory of a delinquent profile or that they are subjects without empathy. For this reason, it appears important to remember that their ideological adherence was only followed by an actual act of violence only in the case of one adolescent girl, whereas two other adolescents changed their minds before acting.

These observations invite us to consider the dimension of relational hold they were subjected to, even if there is no doubt regarding their ideological adherence at the moment of their arrest. Additionally, the altruistic motivations they mostly mention (saving the Syrian population) can also be questioned (10, 11). The results clearly show that these adolescents have maintained their capacity for empathy and that their radical commitment cannot be explained by a lack of empathy. The qualitative analysis allows some speculations regarding the purpose and meaning of life for AMT youths who could expressed stable views. If engagement appeared mainly relational with the group (e.g., being included in the group appears to be a response for feelings of loneliness and marginalisation, for experience of bullying), an idealistic dimension also occurred such as belonging to an ideal society and advocating a grandiose and unique destiny. In addition, at the time of radical engagement, females seemed more engaged through an affective and emotional dimension, whereas males seemed more engaged through intellectualisation and ideology.

In addition, the results of the AMT and CEC group comparisons clearly showed that both populations presented very different profiles. Family environment difficulties of CEC adolescents seem to be more important due to the more regular absence of family members who appear to be less attentive to the adolescents' suffering. It is shown by more frequent absences of a biological parent, more frequent imprisonments of household members, and less frequent resort to care services by families, while psychiatric disorders are more frequent in CEC adolescents. Although the reported data concerning adolescents with radicalised AMT are unique, those on adolescents with CEC are consistent with international reported data, as many studies report high rates of psychiatric disorders, especially conduct disorders (37, 38). We also find very high rates (up to 90%) of imprisoned adolescents with a large predominance of externalised disorders, in particular conduct disorders (39). In addition, we observed frequent precocious and lasting familial dysfunctions, as well as exposure to repetitive traumas and maltreatment (40).

A second striking observation of the AMT prosecuted adolescents is that they have better resources to face their difficulties. These resources can be seen as a result of better academic integration, more effective investment of functional intellectual abilities, a less important level of alienation from family and friends, lower levels of impulsiveness and hostility, and less frequent actual acts (Table 2). Coping strategies used by AMT groups are, for the most part, better than CEC adolescents' and show greater insight (33). Indeed, CEC adolescents more often use tension reduction and avoidance, which indicates a more externalised treatment of their difficulties. As said previously, it is also important to note that AMT prosecuted adolescents present better empathy abilities than CEC adolescents (26). Finally, the main difference between these groups is the predominance of delinquent acts in CEF adolescents, while such acts are committed by a minority of AMT adolescents.

The remaining unresolved question is how to understand radicalised adolescents who carry out their acts (or try to) compared to radicalised adolescents who do not make such attempts (or don't have this intention)? In other words, do radicalised adolescents reported in national files (12) have a particular profile? The widest French cohort from CPDSI (“*Centre de Prévention des Dérives Sectaires liées à l'Islam*,” which is a French deradicalisation programme) evaluated the trajectory and the two-year follow-up of 150 radicalised young people (11, 41). Using a mixed methodology (qualitative and quantitative), two predicting future trajectories appeared. The first concerns young people for whom developmental matters are at the forefront (e.g., individuation, depressive fragility, identity uncertainty) and who, at the beginning, almost exclusively use social networks. This trajectory is more frequent in converts to Islam and contains many family issues, but it is more receptive to educational and psychological interventions (11, 41). The second trajectory, more frequently associated with an assumed violent radicalisation, is part of a close, or neighbourhood, fertile ground with individuals particularly vulnerable to external influence or who have already been enrolled by a religious radical (11, 41). Concerning common variables between the AMT radicalised group of this study and the CPDSI group including 150 subjects, we find one significant variable on the 14 available: the previous imprisonment of a household member (more frequent in the AMT group) (cf. Supplementary Material). The importance of this variable tends to classify adolescents with AMT in the second trajectory (11, 41). In other words, the current results seem to confirm that from the two trajectories evidenced through multivariate modelling in the CPDSI large study (11), the so-called neighbourhood trajectory is the one that shows the worst prognosis.

### Limits

The main limitation of this study is the disparity between AMT and CEC samples. However, it seems important to remember that this phenomenon is extremely marginal, making our AMT group a representative sample (15 of 31 adolescents). Additionally, we took care to verify that the other 16 adolescents did not present significant differences in terms of age, gender, sibling composition, the proportion of school years repeated, the intervention by social services or care pathway. Despite this representativity, a statistical comparison was affected by the disparity between the AMT and CEC groups in terms of number of subjects and age (AMT group is older), but above all, in terms of gender variability, as female subjects represent 40% of the AMT sample but only 5% of the CEF sample. Beyond the observation that the radicalisation phenomenon concerns more young girls (11) than the delinquency phenomenon (42), it seems necessary to consider it a limit for statistical comparison. Another limitation concerns the quick transformation of the radicalisation phenomenon at the time we consider these results (3, 13). Indeed, all 15 AMT prosecuted adolescents examined in this study clearly joined Daesh ideology between 2014 and 2017. Most of them were prosecuted because of conversations with Daesh members (on the internet or in person) that led some of them to try to commit violent acts in France or to travel to the Iraqi-Syrian war zone. Daesh defeat and the reinforcement of French security service operations now restrict the possibility of carrying out such acts, whether due to the drastic reduction of Daesh recruitment campaigns on the internet or to the recent impossibility of joining a territory that is less and less controlled by the jihadist organisation. These contextual elements, decisive for the evolution of radicalisation, raise questions about the reproducibility of our study.

## Conclusion

The results of our study show that AMT prosecuted adolescents are not just delinquents who met an ideology that allows them to legitimate a pre-existing predatory logic. They are adolescents with distinct characteristics. Most of them do not exhibit one or more specific psychiatric disorders, they do not particularly lack empathy, and they are not suicidal adolescents looking for a significant death. Therefore, it is important to consider these characteristics while designating policies to prevent radicalisation, whether through primary prevention in the general population, or through secondary prevention by taking care of AMT prosecuted adolescents. The psychological resources they show through their coping strategies, intellectual skills, capacity for insight, or quest for spirituality allow us to expect positive effects of psychotherapeutic and educational follow-ups.

## Data Availability Statement

The original contributions presented in the study are included in the article/Supplementary Material, further inquiries can be directed to the corresponding author.

## Ethics Statement

The studies involving human participants were reviewed and approved by Comité de Protection des Personnes EST IV, 1 Place de l'hôpital, 67091 STRASBOURG. Written informed consent to participate in this study was provided by the participants' legal guardian/next of kin.

## Author Contributions

GB, DC, and PG contributed to conception and design of the study. NC, ID, AV, and M-AP met and included subjects in the study. ID, HP, LB, and NC organised the database. HP and LB performed the statistical analysis. GB, NC, and DC wrote the first draft of the manuscript. HP wrote a section of the manuscript. All authors contributed to manuscript revision, read, and approved the version we are going to submit.

## Funding

This work was supported by Agence Régionale de Santé de Bretagne, Agence Régionale de Normandie, Centre Hospitalier Universitaire de Brest, Centre Hospitalier Universitaire de Rouen, Hôpitaux Universitaires Pitié-Salpêtrière. The Protection Judiciaire de la Jeunesse mobilised its profesionals for the conduct of the research, and Groupe SOS allowed one of their doctoral students to participate actively in the research.

## Conflict of Interest

The authors declare that the research was conducted in the absence of any commercial or financial relationships that could be construed as a potential conflict of interest.

## Publisher's Note

All claims expressed in this article are solely those of the authors and do not necessarily represent those of their affiliated organizations, or those of the publisher, the editors and the reviewers. Any product that may be evaluated in this article, or claim that may be made by its manufacturer, is not guaranteed or endorsed by the publisher.
