# Supplementary material for: Adolescents Engaged in Radicalisation and Terrorism: A Dimensional and Categorical Assessment
Source: Front Psychiatry. 2022 Jan 14;12:774063. doi: 10.3389/fpsyt.2021.774063 (PMC8795583; doi:10.3389/fpsyt.2021.774063)
Supplement: Supplementary file 1 [file Data_Sheet_1.docx]

**SUPPLEMENT MATERIAL**

**Adolescents engaged in radicalization and terrorism: a dimensional and categorical assessment**

**1. List and localization of the *Centres Educatifs Fermés* that contributed to the recruitment of non-radicalized adolescents with delinquent acts**

**
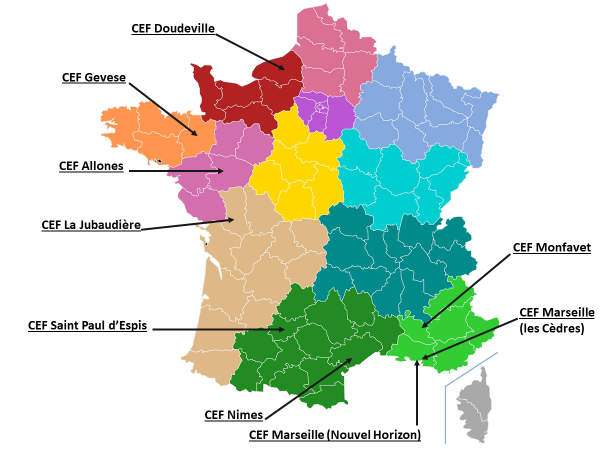
**

**2. Grid used to collect specific characteristics of radicalized individuals condemned for AMT**

| **Rating grids** | | | | | | | | | | |  |
| --- | --- | --- | --- | --- | --- | --- | --- | --- | --- | --- | --- |
|  |  |  |  |  |  |  |  |  |  |  |  |
| **Inclusion number:** | | |  | |  | |  | |  | |  |
| **Date:** |  |  | |  | |  | |  | |  | |
| **Year of birth:** | | |  | |  | |  | |  | |  |
|  |  |  | |  | |  | |  | |  | |
| Radical  acts | Acts told by the subject |  | | | | | | | |  | |
|  | Acts prosecuted by justice |  | | | | | | | |  | |
|  |  |  | |  | |  | |  | |  | |
| **Criteria** | **Characteristics** | **Rating** | | | | | | **Commentary** | |  | |
|  |  | **Score** (Tick with an "X" if unexploitable) | | | | **Refusal** (Tick with an "X") | |  |  |  | |
| **Sociocultural characteristics** | Boy (1) / Girl (0) |  | | | |  | |  | |  | |
|  | Religious denomination (0 Without denomination, 1 Islam, 2 Christianism, 3 Judaism, 4 Hinduism, 5 Buddhism, 6 Others) |  | | | |  | |  | |  | |
|  | Familial religious denomination  (0 Without denomination, 1 Islam, 2 Christianism, 3 Judaism, 4 Hinduism, 5 Buddhism, 6 Others) |  | | | |  | |  | |  | |
|  | Conversion to Islam (0 No, 1 Yes) |  | |  | |  | |  | |  | |
|  | Family socioeconomic insecurity |  | | | |  | |  | |  | |
|  |  |  | |  | |  | |  | |  | |
| **Criteria** | **Characteristic** | **Score** (0 No, 1 Yes) (Tick with an "X" if unexploitable) | | | | **Refusal** (Tick with an "X") | | **Commentary** | |  | |
|  |  |  |  |  |  |  |  |  |  |  | |
| **Life history (Before radical commitment)** | Young parent with early age child |  | | | |  | |  | |  | |
|  | Professional projects related to assistance/ care/humanitarian aid |  | | | |  | |  | |  | |
|  | Life history sexual abuse (clarify if domestic or extra-familial) |  | | | |  | |  | |  | |
|  | Family member sudden traumatic event (bereavement, illness, separation) |  | | | |  | |  | |  | |
|  | Domestic violence |  | |  | |  | |  | |  | |
|  | Academic or professional multiple discontinuities |  | |  | |  | |  | |  | |
|  | Be refused after army and/or police entrance examination |  | | | |  | |  | |  | |
|  | Absent father |  | |  | |  | |  | |  | |
|  | Absent mother |  | | | |  | |  | |  | |
|  | Known by child welfare services |  | | | |  | |  | |  | |
|  | Placed in children’s home or foster family |  | |  | |  | |  | |  | |
|  |  |  | |  | |  | |  | |  | |
| **Criteria** | **Characteristic** | **Score** (0 No, 1 Yes) (Tick with an "X" if unexploitable) | | | | **Refusal** (Tick with an "X") | | **Commentary** | |  | |
|  |  |  |  |  |  |  |  |  |  |  | |
| **Life history (Before radical commitment)** | Educational action in an open setting |  | | | |  | |  | |  | |
|  | Hospitalisation in a child psychiatry unit |  | | | |  | |  | |  | |
|  | Follow-up by a child psychiatrist |  | | | |  | |  | |  | |
|  | Suicidal attempt in their life course |  | | | |  | |  | |  | |
|  | Psychotherapy follow-up/Psychological Consultations |  | |  | |  | |  | |  | |
|  | Political/Civic commitment |  | | | |  | |  | |  | |
|  | Sentence for violence against people |  | |  | |  | |  | |  | |
|  | Sentence for drug trafficking |  | | | |  | |  | |  | |
|  | Sentence for another traffic |  | | | |  | |  | |  | |
|  | Sentence for damage to property |  | |  | |  | |  | |  | |
|  |  |  | |  | |  | |  | |  | |
|  |  |  | |  | |  | |  | |  | |
| **Criteria** | **Characteristic** | **Score** (0 No, 1 Yes) (Tick with an "X" if unexploitable) | | | | **Refusal** (Tick with an "X") | | **Commentary** | |  | |
|  |  | **Preliminary** | | **After radic.** | |  |  |  |  |  | |
| **Symptomatology, Psychopathology and Behaviours** | Obsessive-compulsive disorder |  | |  | |  | |  | |  | |
|  | Over-invested religious rituals |  | |  | |  | |  | |  | |
|  | Sexual obsessions |  | |  | |  | |  | |  | |
|  | Very sexualised appearance |  | |  | |  | |  | |  | |
|  | Sexual risk behaviour |  | |  | |  | |  | |  | |
|  | Drug addiction/Use of psychoactive substances |  | |  | |  | |  | |  | |
|  | Video game addiction |  | |  | |  | |  | |  | |
|  | Addiction behaviours (tobacco excluded) |  | |  | |  | |  | |  | |
|  | Looking for security in the group |  | |  | |  | |  | |  | |
|  |  |  | |  | |  | |  | |  | |
|  |  |  | |  | |  | |  | |  | |
| **Criteria** | **Characteristic** | **Score** (0 No, 1 Yes) (Tick with an "X" if unexploitable) | | | | **Refusal** (Tick with an "X") | | **Commentary** | |  | |
|  |  | **Preliminary** | | **After radic.** | |  |  |  |  |  | |
| **Symptomatology, Psychopathology and Behaviours** | Suicidal wishes |  | |  | |  | |  | |  | |
|  | Risky behaviours |  | |  | |  | |  | |  | |
|  | Scarification |  | |  | |  | |  | |  | |
|  | Eating disorders |  | |  | |  | |  | |  | |
|  | Sleep disorders |  | |  | |  | |  | |  | |
|  | Repetitive runaway |  | |  | |  | |  | |  | |
|  | Vagrancy |  | |  | |  | |  | |  | |
|  | Delinquent behaviour |  | |  | |  | |  | |  | |
|  | Violent behaviour towards others |  | |  | |  | |  | |  | |
|  |  |  | |  | |  | |  | |  | |
| **Criteria** | **Characteristic** | **Score** (0 No, 1 Yes) (Tick with an "X" if unexploitable) | | | | **Refusal** (Tick with an "X") | | **Commentary** | |  | |
|  |  |  |  |  |  |  |  |  |  |  | |
| **Visited internet videos** | Viewing of preaching videos |  | | | |  | |  | |  | |
|  | Viewing of macabre/dehumanisation videos |  | | | |  | |  | |  | |
|  | Viewing of unbearable war videos |  | | | |  | |  | |  | |
|  | Viewing of idealisation propaganda videos |  | | | |  | |  | |  | |
|  | Alternation with pornographic videos viewing |  | | | |  | |  | |  | |
| **Commitment reasons** | Desire of escaping from living environment |  | | | |  | |  | |  | |
|  | Searching for an ideal |  | | | |  | |  | |  | |
|  | Feeling of discrimination/injustice |  | | | |  | |  | |  | |
|  | Altruistic speech |  | | | |  | |  | |  | |
|  | Fear of hell/imminent end of the world |  | | | |  | |  | |  | |
|  | Adrenaline searching |  | |  | |  | |  | |  | |
|  | Armed struggle attraction |  | | | |  | |  | |  | |
|  | Aspirations of others domination |  | | | |  | |  | |  | |
|  | Searching for clear sexual identity |  | | | |  | |  | |  | |
|  |  |  | |  | |  | |  | |  | |
| **Criteria** | **Characteristic** | **Score** (0 No, 1 Yes) (Tick with an "X" if unexploitable) | | | | **Refusal** (Tick with an "X") | | **Commentary** | |  | |
|  |  |  |  |  |  |  |  |  |  |  | |
| **Reported background links with radicalisation** | Feeling of being rejected |  | | | |  | |  | |  | |
|  | Feeling of injustice |  | | | |  | |  | |  | |
|  | Humiliation experiences |  | | | |  | |  | |  | |
|  | Feeling of being from nowhere/of non-belonging |  | |  | |  | |  | |  | |
|  | Express death wishes |  | | | |  | |  | |  | |
| **Speech during interview** | Manipulative speech |  | | | |  | |  | |  | |
|  | Seductive speech |  | | | |  | |  | |  | |
|  | Speech without affectivity |  | | | |  | |  | |  | |
|  | Restrictive speech |  | | | |  | |  | |  | |
|  | Aggressive speech |  | |  | |  | |  | |  | |
|  | Denigrating speech |  | |  | |  | |  | |  | |
|  | Reluctant speech |  | | | |  | |  | |  | |
|  | Express death wishes |  | | | |  | |  | |  | |
|  |  |  | |  | |  | |  | |  | |
| **Criteria** | **Characteristic** | **Score** (0 No, 1 Yes) (Tick with an "X" if unexploitable) | | | | **Refusal** (Tick with an "X") | | **Commentary** | |  | |
|  |  |  |  |  |  |  |  |  |  |  | |
| **Group dimension** | Solitary radicalisation |  | | | |  | |  | |  | |
|  | Group radicalisation |  | | | |  | |  | |  | |
|  | Initial contact with radicalised individuals on the internet |  | | | |  | |  | |  | |
|  | Initial contact in person |  | | | |  | |  | |  | |
|  | Mention of a “mentor” figure |  | | | |  | |  | |  | |
|  |  |  | |  | |  | |  | |  | |

**3. Comparison between the AMT group and the CIPDR sample (Campelo et al., 2018)**

Only binary variables were common in the two studies. Given the differences in age and gender between the two groups, we used logistic regressions adjusted for age and gender to compare the common variables between the two samples

| **Age and gender in the AMT group and the CIPDR sample** | | |
| --- | --- | --- |
|  | AMT group (N=15) | CIPDR sample (150) |
| Gender (F/M) | 6 (40%) / 9 (60%) | 101 (67%) / 49 (33%) |
| Age: mean (SD) [min-max] | 16.93 (1.03) [14-18] | 19.82 (5.28) [13-40] |

## Binary variables

Formula: variable ~ group + age + gender

Versus CIPDR

| Variable | Estimate_AMT | OR_AMT | 95%CI_OR_AMT | p_value_AMT | N |
| --- | --- | --- | --- | --- | --- |
| Living with both parents | -1.02 | 0.36 | [0.08;1.25] | 0.138 | 165 |
| Loss of a biological parent | -0.91 | 0.4 | [0.05;1.88] | 0.295 | 81 |
| Parental history of medical condition | -0.63 | 0.53 | [0.11;1.87] | 0.363 | 165 |
| History of medical condition | -0.7 | 0.5 | [0.07;2.06] | 0.39 | 165 |
| History of educational support | 0.96 | 2.61 | [0.81;10.11] | 0.128 | 165 |
| History of psychiatric hospitalization | 0.55 | 1.73 | [0.35;6.58] | 0.449 | 165 |
| History of psychiatric treatment | -0.22 | 0.8 | [0.24;2.48] | 0.71 | 165 |
| History of abuse | -1.29 | 0.28 | [0.01;1.63] | 0.236 | 165 |
| History of physical violence | 1.06 | 2.89 | [0.85;9.55] | 0.082 . | 165 |
| History of psychological violence | -0.89 | 0.41 | [0.12;1.55] | 0.167 | 164 |
| Family history of addiction | -0.98 | 0.38 | [0.06;1.52] | 0.224 | 164 |
| **Family history of imprisonment** | **1.47** | **4.35** | **[1.01;17.08]** | **0.037 *** | **164** |
| Physical contact with a radicalized individual | -1 | 0.37 | [0.09;1.17] | 0.109 | 165 |
| Family socio-economic insecurity | 0.26 | 1.3 | [0.37;4.09] | 0.659 | 165 |
